# Elevated C-reactive protein level during clinical remission can predict poor outcomes in patients with Crohn’s disease

**DOI:** 10.1371/journal.pone.0179266

**Published:** 2017-06-16

**Authors:** Kyunghwan Oh, Eun Hye Oh, Seunghee Baek, Eun Mi Song, Gwang-Un Kim, Myeongsook Seo, Sung Wook Hwang, Sang Hyoung Park, Dong-Hoon Yang, Kyung-Jo Kim, Jeong-Sik Byeon, Seung-Jae Myung, Suk-Kyun Yang, Byong Duk Ye

**Affiliations:** 1 Department of Internal Medicine, University of Ulsan College of Medicine, Asan Medical Center, Seoul, Korea; 2 Department of Gastroenterology, University of Ulsan College of Medicine, Asan Medical Center, Seoul, Korea; 3 Department of Clinical Epidemiology and Biostatistics, University of Ulsan College of Medicine, Asan Medical Center, Seoul, Korea; 4 Inflammatory Bowel Disease Center, University of Ulsan College of Medicine, Asan Medical Center, Seoul, Korea; University Hospital Llandough, UNITED KINGDOM

## Abstract

Intestinal inflammation and mucosal damage in Crohn’s disease (CD) are believed to progress even during clinical remission. We investigated the long-term prognosis of CD patients in clinical remission according to serum C-reactive protein (CRP) levels. This study included 339 CD patients in clinical remission (Crohn’s disease activity index < 150) for more than 6 months between January 2008 and December 2010. Clinical outcomes were compared between patients with normal and elevated CRP levels during clinical remission. During clinical remission, 150 patients had normal CRP consistently and 189 had elevated CRP at least once. During follow-up (median, 7.9 years [interquartile range, 6.8–8.0]), the Kaplan–Meier analysis with the log-rank test showed that normal CRP group had a longer CD-related hospitalization-free survival (P = 0.007) and a longer CD-related intestinal resection-free survival (P = 0.046) than elevated CRP group. In multivariate analysis, elevated CRP was significantly and independently associated with an increased risk of subsequent CD-related hospitalization (adjusted hazard ratio [aHR] 1.787, 95% confidence interval [CI]: 1.245–2.565, P = 0.002) and of subsequent CD-related intestinal resection (aHR 1.726, 95% CI: 1.003–2.969, P = 0.049). The most common reason for CD-related hospitalization was penetrating complications (35.6%). Even when CD patients are in clinical remission, elevated CRP is significantly associated with subsequent CD-related hospitalization and CD-related intestinal resection during follow-up. CD patients in clinical remission but elevated CRP should receive more careful attention and timely interventions to improve long-term outcomes.

## Introduction

Crohn’s disease (CD) is among the major types of inflammatory bowel diseases (IBD) that affect the whole gastrointestinal tract, and its global incidence and prevalence are increasing [1–3]. Most patients with CD experience a waxing and waning clinical course of relapse and remission and develop cumulative structural damage to the bowel over time [4]. It is believed that a preclinical stage of CD exists before the development of clinical symptoms and that inflammation begins before the clinical diagnosis of CD, resulting in progressive bowel damage even in the preclinical stage [4]. Therefore, effective medical treatment to stop or slow the progression of bowel damage is critical, and early, aggressive therapy is recommended to prevent major complications such as bowel stricture or perforation [5]. The identification of risk factors for disease progression in CD patients is crucial for the individualization of appropriate therapy [4].

Historically, the Crohn’s disease activity index (CDAI), which is based primarily on subjective symptoms, has been widely used in the medical management of CD [6]. However, CDAI scores reportedly have poor correlation with both inflammatory biomarkers such as C-reactive protein (CRP) and endoscopic disease activity [7–9]. Therefore, there may be a subgroup of CD patients who despite being in clinical remission, have a high burden of bowel inflammation and a high probability of progressive bowel damage.

Recently, a study by Click et al. reported that asymptomatic CD patients with elevated CRP levels had a risk of hospitalization higher than that in patients with normal CRP levels [10]. However, the study was limited because CRP levels were measured only once at enrollment and only a small number of CD patients with elevated CRP were included [10]. Moreover, observations among CD patients in Western countries may not apply directly to Asian patients owing to differences in genetic background, clinical characteristics, and long-term prognosis [11]. Therefore, we tested the hypothesis that Asian CD patients in clinical remission who have high CRP levels show poorer long-term prognosis compared with that of similar patients with normal CRP levels.

## Materials and methods

### Study population

Patients with confirmed CD who were prospectively enrolled in the IBD registry of Asan Medical Center, a tertiary care center in Korea, were included. The diagnosis of CD was based on conventional clinical, radiologic, endoscopic, and histopathologic criteria [12–14]. The characteristics of the IBD registry are previously described in detail [15]. Eligible patients were defined as those who visited the clinic at least twice within 6 months between January 2008 and December 2010, and had records of same-day measurements of CDAI and CRP level for each clinic visit. All patients with confirmed CD in our hospital were instructed via demonstration to record their symptoms during 7 days before a clinic visit to calculate CDAI. Complete blood count, serum chemistry, and CRP were also checked on the day of the visit. At every visit, CDAI score was calculated using a computerized program incorporated into our electronic medical record system, and the value was recorded in the clinical note. Patients without CDAI or CRP data and those with ileostomies or colostomies were excluded from the study.

**Fig 1:**
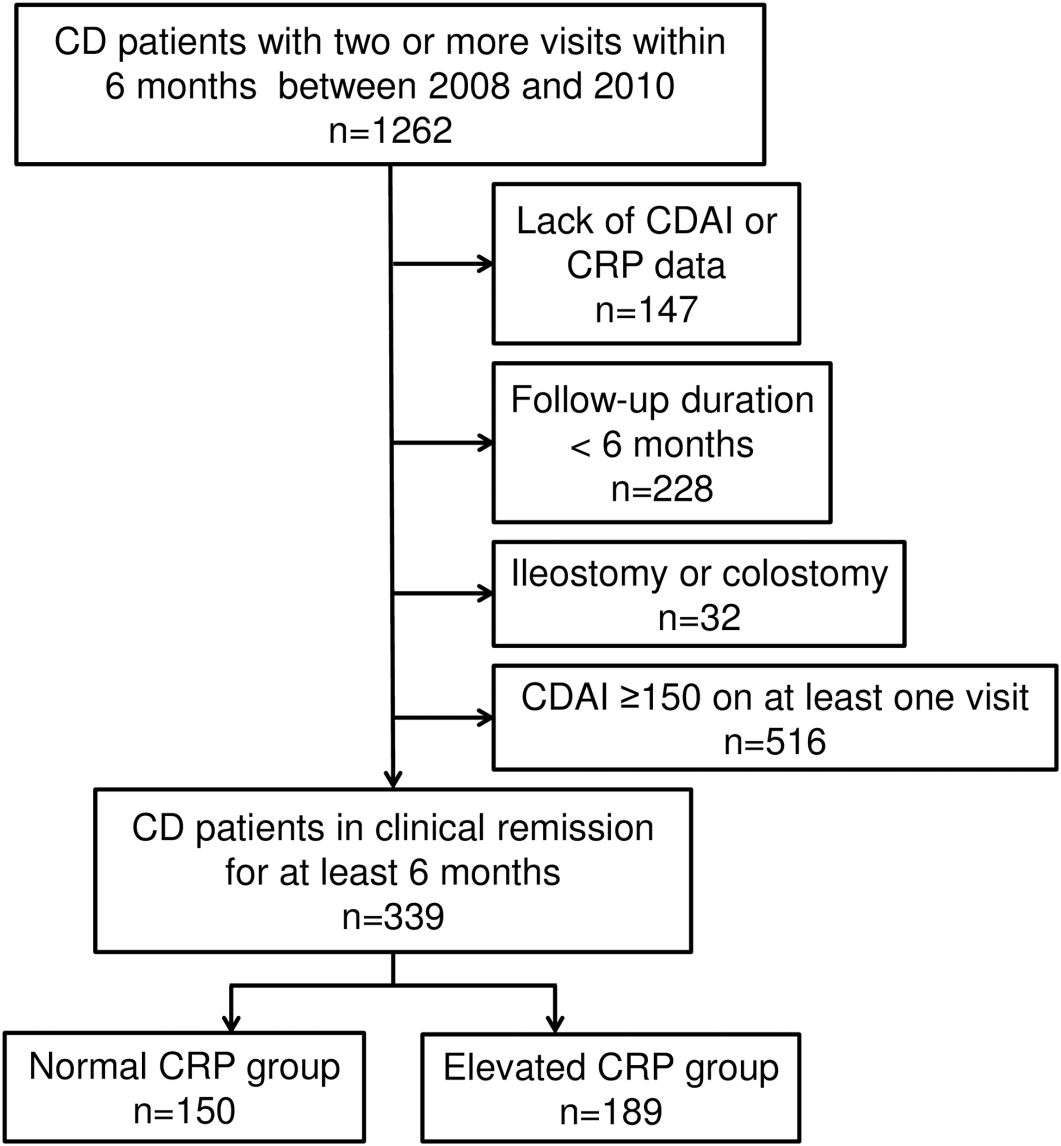
Study subjects. CD, Crohn’s disease; CDAI, Crohn’s disease activity index; CRP, C-reactive protein.

Among 1262 eligible subjects, 147 subjects were excluded due to lack of CDAI or CRP data, 228 due to inadequate length of follow-up (less than 6 months), and 32 due to having an ileostomy or colostomy (Fig 1). Of the remaining 855 subjects, 516 had CDAI values of ≥150 on at least one visit within 6 months (Fig 1). Therefore, a total of 339 patients with CD in clinical remission for at least 6 months were enrolled (Fig 1). The study population was divided into patients with elevated CRP levels and those with normal CRP levels (Fig 1).

Patients in the elevated CRP group showed elevated CRP levels on one or more clinic visits, whereas patients in the normal CRP group showed normal CRP at all visits for 6 months.

The upper limit of CRP at our laboratory was 0.6 mg/dL and a CRP level of > 0.6 mg/dL was defined as elevated. CRP was measured with latex particle–enhanced immunoturbidimetric assay using the Cobas^®^ 8000 c702 Chemistry Autoanalyzer (Roche Diagnostic International Ltd, Rotkreuz ZG, Switzerland). Baseline characteristics such as age, sex, height, weight, smoking status, family history of IBD, comorbidities, disease duration, Montreal disease location and behavior [16], extraintestinal manifestations (arthralgia/arthritis, uveitis, erythema nodosum, pyoderma gangrenosum, and oral aphthous ulcers), history of CD-related surgery, and history of perianal surgery were collected. Medication data including use of 5-aminosalicylic acids, systemic corticosteroids, immunomodulators (azathioprine, 6-mercaptopurine, and methotrexate), and anti-tumor necrosis factor-α (TNF-α) agents (infliximab and adalimumab) were also collected.

### Outcome variables

Clinical follow-up data were collected retrospectively from the last clinic visit during the 6 months of CDAI and CRP measurement. Our treatment protocol for CD patients was described in detail elsewhere [15].

The co-primary outcome variables were CD-related hospitalization and CD-related intestinal resection during follow-up. CD-related hospitalization was confirmed through the detailed review of all admission records, including admission notes and discharge summaries. Admissions for non CD-related reasons or solely for the injection of infliximab were excluded. CD-related intestinal resection was confirmed through detailed review of operative notes and discharge summaries.

The co-secondary outcome variables were new perianal surgery, new CD-related intestinal complications, and step-up of CD medication. New perianal surgery (perianal abscess incision and drainage, seton ligation, fistulotomy, modified Hanley procedure, and stem cell implantation) was also confirmed through the detailed review of operative notes and discharge summaries. New CD-related intestinal complications were defined as both new Montreal stricturing (B2) or penetrating (B3) complications in patients with B1 behaviors at the beginning of follow-up and new Montreal B3 complications in patients with B2 behaviors at the beginning of follow-up. Step-up of medication was defined as the addition of immunomodulators or anti-TNF-α agents, or both, in 5-aminosalicylic acid agent users and the addition of anti-TNF-α agents in immunomodulator users during follow-up.

### Statistical analysis

Continuous variables were expressed as means with standard deviation or medians with interquartile range (IQR) as appropriate. Categorical variables were expressed as proportions and percentages. Continuous variables were compared using the Student’s t-test or the Mann–Whitney U test. Categorical variables were compared using Pearson’s chi-squared test or Fisher’s exact test as appropriate. The cumulative probabilities of developing outcome events were calculated using the Kaplan–Meier method and compared between the elevated CRP and normal CRP groups using the log-rank test.

To identify factors predictive of CD-related hospitalization and intestinal resection, we performed multivariate Cox regression analysis. All variables with *P* values of < 0.2 from univariate Cox regression analysis were included in the multivariate model.

All tests were two-sided with the statistical significance set at a *P* value of 0.05. Analyses were performed with SPSS statistical software, version 23.0 for Windows (IBM, New York, NY, USA).

### Ethical considerations

The study protocol was approved and a waiver of consents from study subjects was granted by the Institutional Review Board of Asan Medical Center (IRB No. 2016–0995).

## Results

### Baseline characteristics

The median number of clinic visits over 6 months was 4 (IQR 3–6) in a total of 339 patients. We found no significant differences in age, sex, smoking status, family history of IBD, Montreal location and behavior of CD, extraintestinal manifestations, medications used, or history of bowel or perianal surgery between the elevated CRP group (n = 189) and the normal CRP group (n = 150; Table 1). Disease duration in the normal CRP group (median, 4.1 years [IQR, 2.1–7.1]) was longer than that in the elevated CRP group (median, 3.0 years [IQR, 1.3–6.1], *P* = 0.022). Median CRP at the beginning of follow-up was 0.1 mg/dL (range, 0.03–0.6) in the normal CRP group and 0.82 mg/dL (range, 0.05–8.91) in the elevated CRP group (*P* < 0.001).

**Table 1:** Baseline characteristics at the beginning of follow-up.

	Total (n = 339)	Normal CRP group (n = 150)	Elevated CRP group (n = 189)	*P* value
Age at CD diagnosis, median (IQR), years	27 (22–35)	27 (23–35)	26 (22–34)	0.907^c^
Montreal classification of age at CD diagnosis, years				0.366^d^
A1 (≤16) (%)	21 (6.2)	9 (6.0)	12 (6.4)	
A2 (17–39) (%)	303 (89.4)	137 (91.3)	166 (87.8)	
A3 (≥40) (%)	15 (4.4)	4 (2.7)	11 (5.8)	
Male (%)	254 (74.9)	110 (73.3)	144 (76.2)	0.547^d^
Smoking at CD diagnosis (%)^a^				0.402^d^
Never	227 (67.2)	95 (63.3)	132 (70.2)	
Former	25 (7.4)	12 (8.0)	13 (6.9)	
Current	86 (25.4)	43 (28.7)	43 (22.9)	
Family history of IBD at CD diagnosis (%)	24 (7.1)	13 (8.7)	11 (5.8)	0.310^d^
Disease duration, median (IQR), years	3.3 (1.8–6.4)	4.1 (2.1–7.1)	3.0 (1.3–6.1)	0.007^c^
Height, mean (SD), cm^b^	169.6 (8.16)	169.1 (8.11)	170.0 (8.21)	0.381^e^
Weight, mean (SD), kg^b^	57.03 (12.11)	56.52 (12.29)	57.49 (11.98)	0.509^e^
CDAI, median (IQR)	54.59 (33.7–80.84)	50.25 (28.14–79.10)	58.08 (38.34–82.39)	0.043^c^
CRP, median (range), mg/dL	0.31 (0.03–8.91)	0.1 (0.03–0.6)	0.82 (0.05–8.91)	<0.001^c^
Montreal location (%)				0.408 ^d^
Ileal (L1)	73 (21.5)	35 (23.3)	38 (20.1)	
Colonic (L2)	24 (7.1)	13 (8.7)	11 (5.8)	
Ileocolonic (L3)	242 (71.4)	102 (68.0)	140 (74.1)	
Montreal behavior (%)				
Non-stricturing, non-penetrating (B1)	177 (52.2)	77 (51.3)	100 (52.9)	0.659 ^d^
Stricturing (B2)	50 (14.8)	20 (13.4)	30 (15.8)	
Penetrating (B3)	112 (33.0)	53 (35.3)	59 (31.3)	
Perianal disease modifier (p)	183 (54.0)	85 (56.7)	98 (51.9)	0.377 ^d^
Extraintestinal manifestations (%)				
Arthralgia/Arthritis	54 (15.9)	23 (15.3)	31 (16.4)	0.870 ^d^
Uveitis	0 (0)	0 (0)	0 (0)	NA
Erythema nodosum	5 (1.5)	3 (2.0)	2 (1.1)	0.456 ^d^
Pyoderma gangrenosum	2 (0.6)	2 (1.3)	0 (0)	0.107 ^d^
Oral aphthous ulcers	35 (10.3)	15 (10.0)	20 (10.6)	0.925 ^d^
Medications (%)				0.918 ^d^
5-ASA only	108 (31.9)	50(33.3)	58 (30.7)	
Immunomodulators	208 (61.3)	90(60.0)	118 (62.4)	
Anti TNF-α agents	23 (6.8)	10 (6.7)	13 (6.9)	
Past history of intestinal resection (%)	92 (27.1)	43 (28.7)	49 (25.9)	0.573 ^d^
Past history of perianal surgery (%)	163 (48.1)	76 (50.7)	87 (46.0)	0.396 ^d^

5-ASA, 5-aminosalicylic acid; CD, Crohn’s disease; CDAI, Crohn’s disease activity index; CRP, C-reactive protein; IBD, inflammatory bowel disease; IQR, interquartile range; NA, not applicable; SD, standard deviation; TNF-α, tumor necrosis factor-α

^a^Missing data on 1 patient in elevated CRP group.

^b^Missing data on 64 patients (20 patients in normal CRP group, 44 patients in elevated CRP group).

We used ^c^Mann–Whitney U test, ^d^Pearson’s chi-squared test, and ^e^Student’s t-test for statistical analysis.

### Hospitalization during follow-up

**Fig 2:**
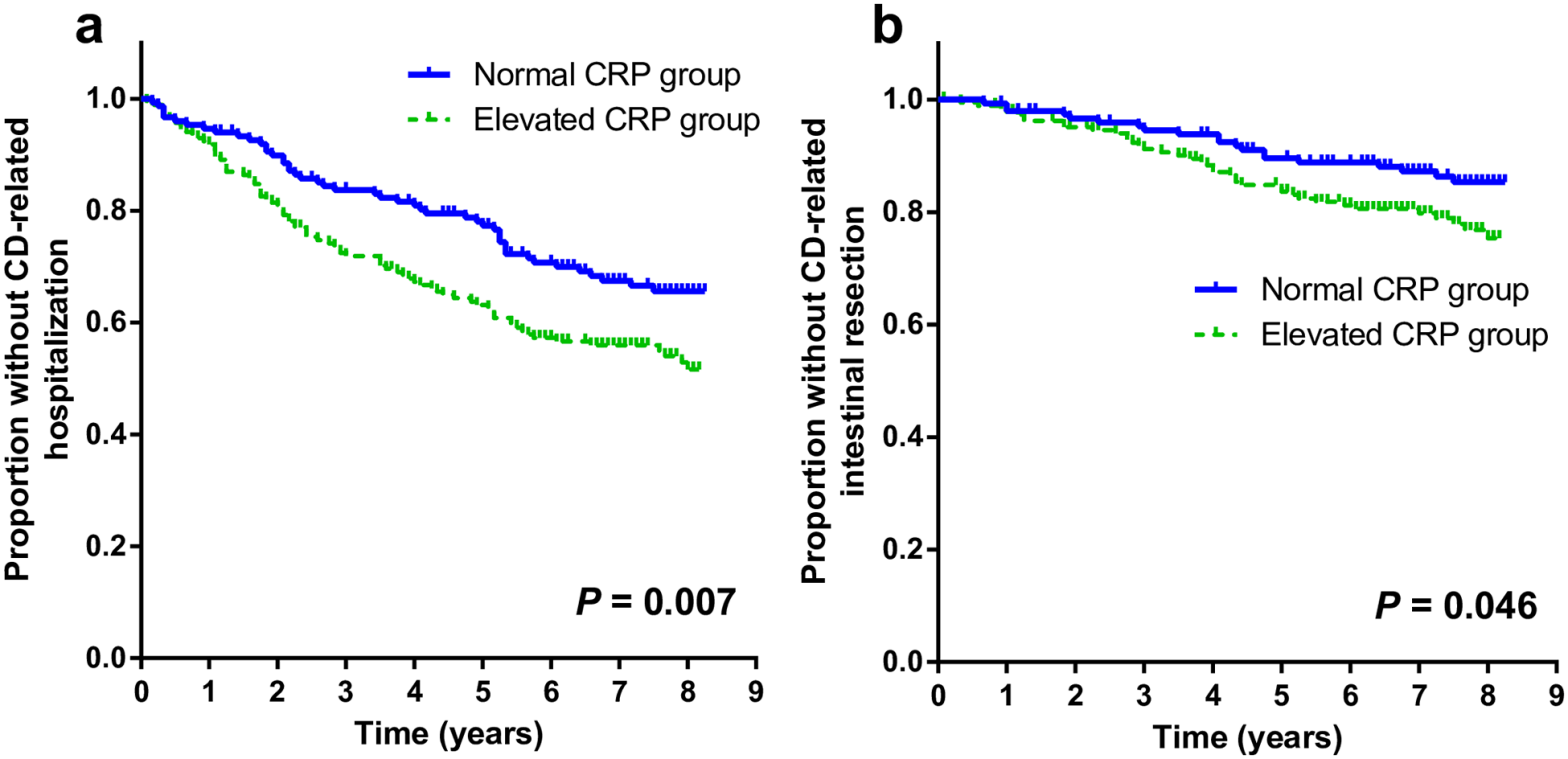
Kaplan–Meier CD-related hospitalization-free survival curves (a) and CD-related intestinal resection–free survival curves (b). CD, Crohn’s disease; CRP, C-reactive protein.

At the final follow-up date (July 31, 2016), 64 patients had been lost to follow-up (26 patients in the normal CRP group [17.3%] and 38 patients in the elevated CRP group [20.1%], *P* = 0.420), and 275 patients were followed to the final date. During the follow-up period (median, 7.9 years [IQR, 6.8–8.0]), more patients with elevated CRP levels were hospitalized than patients with normal CRP (normal CRP group, 48 patients [32.0%] versus elevated CRP group, 84 patients [44.4%], *P* = 0.025; Table 2). Additionally, in the Kaplan-Meier analysis with the log-rank test, elevated CRP group showed a significantly shorter time to hospitalization than normal CRP group (*P* = 0.007; Fig 2a).

**Table 2:** Overall outcome variables and reasons for hospitalization in both groups.

	Total (n = 339)	Normal CRP group (n = 150)	Elevated CRP group (n = 189)	*P* value
Follow-up duration, median (IQR), years	7.9 (6.8–8.0)	8.0 (7.0–8.1)	7.8 (6.5–8.0)	0.008^d^
Intestinal resection (%)	59 (17.4)	20 (13.3)	39 (20.6)	0.085^e^
New perianal surgery (%)^a^	10 (5.7)	2 (2.7)	8 (7.8)	0.195^e^
New complications (%)^b^	52 (26.9)	24 (29.3)	28 (25.2)	0.623^e^
Step-up of medications (%)^c^	144 (45.6)	65 (46.4)	79 (44.9)	0.785^e^
CD-related hospitalization (%)	132 (38.9)	48 (32.0)	84 (44.4)	0.025^e^
Reasons for CD-related hospitalization				0.750^e^
Disease flare (without B2 or B3 complication) (%)	18 (13.6)	7 (14.6)	11 (13.1)	
CD complication				
Stricturing (B2) (%)	29 (22.0)	10 (20.8)	19 (22.6)	
Penetrating (B3) (%)	47 (35.6)	19 (39.6)	28 (33.3)	
Bleeding (%)	14 (10.6)	6 (12.5)	8 (9.5)	
Perianal disease (%)	22 (16.7)	6 (12.5)	16 (19.1)	
Others (%)	2 (1.5)	0 (0)	1 (1.2) (combined CMV colitis)	
			1 (1.2) (further evaluation for suspected ileal neoplasia)	

CD, Crohn’s disease; CMV, cytomegalovirus; CRP, C-reactive protein.

^a^We excluded 163 patients due to past history of perianal surgery (76 from normal CRP group, 87 from elevated CRP group).

^b^We excluded 146 patients due to past history of CD-related complications (68 from normal CRP group, 78 from elevated CRP group).

^c^We excluded 23 patients due to the use of anti-tumor necrosis factor-α agents at the start of follow up (10 from normal CRP group, 13 from elevated CRP group).

We used ^d^Mann–Whitney U test and ^e^Pearson’s chi-squared test for statistical analysis.

The most common reason for hospitalization was Montreal B3 complications in the entire study subjects (35.6%), and in each group (39.6% in normal CRP group and 33.3% in elevated CRP group, Table 2). We found no significant differences between the groups in the reasons for hospitalization (*P* = 0.750).

In the multivariate Cox regression analysis, patients with elevated CRP levels were 1.787 times more likely to be hospitalized (adjusted hazard ratio [aHR] = 1.787; 95% confidence interval [CI]: 1.245–2.565, *P* = 0.002). In addition, patients with Montreal B3 behavior at baseline were 2.175 times more likely to be hospitalized (aHR = 2.175, 95% CI: 1.489–3.177, *P* < 0.001), and patients with Montreal perianal modifier were 1.446 times more likely to be hospitalized (aHR = 1.446, 95% CI: 1.016–2.058, *P* = 0.040; Table 3).

**Table 3:** Univariate and multivariate Cox regression analysis for factors associated with hospitalization.

	Univariate analysis			Multivariate analysis		
	aHR	95% CI	*P*	aHR	95% CI	*P*
Age at CD diagnosis, years						
A1 (≤16)	1.154	0.378–3.529	0.801	Not included		
A2 (17–39)	1.193	0.488–2.920	0.699			
A3 (≥40)	Reference					
Male	0.811	0.556–1.182	0.275	Not included		
Elevated CRP	1.619	1.135–2.310	0.008	1.787	1.245–2.565	0.002
CDAI	1.002	0.997–1.006	0.433	Not included		
Montreal location						
Ileal (L1)	Reference			Not included		
Colonic (L2)	0.609	0.252–1.476	0.273			
Ileocolonic (L3)	1.147	0.749–1.755	0.528			
Montreal behavior						
Non-stricturing, non-penetrating (B1)	Reference			Reference		
Stricturing (B2)	2.099	1.235–3.569	0.006	1.474	0.882–2.461	0.138
Penetrating (B3)	3.636	2.263–5.840	<0.001	2.175	1.489–3.177	<0.001
Perianal disease modifier (p)	1.463	1.031–2.078	0.033	1.446	1.016–2.058	0.040
Smoking at CD diagnosis						
Never	Reference			Not included		
Former	0.790	0.399–1.564	0.498			
Current	0.773	0.507–1.178	0.231			
Family history of IBD at CD diagnosis	1.545	0.871–2.738	0.137	1.591	0.881–2.870	0.123
Medications						
5-ASA only	Reference			Not included		
Immunomodulators	0.858	0.589–1.250	0.426			
Anti TNF-α agents	1.436	0.770–2.676	0.255			
Disease duration	1.000	0.996–1.004	0.948	Not included		
Past history of intestinal resection	1.011	0.694–1.474	0.953	Not included		
Past history of perianal surgery	0.938	0.667–1.319	0.712	Not included		

5-ASA, 5-aminosalicylic acid; aHR, adjusted hazard ratio; CD, Crohn’s disease; CI, confidence interval; CRP, C-reactive protein; IBD, inflammatory bowel disease, TNF-α, tumor necrosis factor-α

### Intestinal resection during follow-up

In the Kaplan–Meier analysis with the log rank test, elevated CRP group showed a significantly shorter time to subsequent intestinal resection than normal CRP group during follow-up (*P* = 0.046; Fig 2b). In the multivariate Cox regression analysis, patients with elevated CRP levels had 1.726 times the risk of CD-related intestinal resection (aHR = 1.726, 95% CI: 1.003–2.969, *P* = 0.049; Table 4). In addition, patients with Montreal B2 or B3 behavior at baseline also had a higher risk of CD-related intestinal resection (B2: aHR = 2.722, 95% CI: 1.223–6.058, *P* = 0.014: B3: aHR = 4.149, 95% CI: 2.177–7.907, *P* < 0.001). Furthermore, compared with patients who had used 5-aminosalicylic acids only, patients who had used immunomodulators (without ever using anti-TNF-α agents) experienced more frequent CD-related intestinal resection during follow-up (aHR = 2.147, 95% CI: 1.076–4.284, *P* = 0.030).

**Table 4:** Univariate and multivariate Cox regression analysis for factors associated with CD-related intestinal resection.

	Univariate analysis			Multivariate analysis		
	aHR	95% CI	*P*	aHR	95% CI	*P*
Age at CD diagnosis, years						
A1 (≤16)	2.277	0.237–21.893	0.476	Not included		
A2 (17–39)	2.997	0.415–21.660	0.277			
A3 (≥40)	Reference					
Male	0.777	0.446–1.352	0.372	Not included		
Elevated CRP	1.720	1.003–2.950	0.049	1.726	1.003–2.969	0.049
CDAI	0.999	0.992–1.006	0.706	Not included		
Montreal location						
Ileal (L1)	Reference			Not included		
Colonic (L2)	<0.001		0.967			
Ileocolonic (L3)	1.203	0.638–2.269	0.567			
Montreal behavior						
Non-stricturing, non-penetrating (B1)	Reference			Reference		
Stricturing (B2)	2.961	1.344–6.523	0.007	2.722	1.223–6.058	0.014
Penetrating (B3)	4.195	2.251–7.820	<0.001	4.149	2.177–7.907	<0.001
Perianal disease modifier (p)	1.198	0.715–2.008	0.493	1.118	0.659–1.898	0.678
Smoking at CD diagnosis						
Never	Reference			Not included		
Former	1.076	0.425–2.722	0.877			
Current	0.881	0.472–1.644	0.691			
Family history of IBD at CD diagnosis	1.413	0.607–3.287	0.422	Not included		
Medications						
5-ASA only	Reference			Reference		
Immunomodulators	2.270	1.142–4.510	0.019	2.147	1.076–4.284	0.030
Anti TNF-α agents	2.293	0.784–6.711	0.130	1.901	0.640–5.647	0.248
Disease duration	1.004	0.998–1.009	0.190	1.000	0.994–1.005	0.916
Past history of intestinal resection	1.285	0.749–2.204	0.362	Not included		
Past history of perianal surgery	0.895	0.536–1.495	0.672	Not included		

5-ASA, 5-aminosalicylic acid; aHR, adjusted hazard ratio; CD, Crohn’s disease; CI, confidence interval; CRP, C-reactive protein; IBD, inflammatory bowel disease; TNF-α, tumor necrosis factor-α

### New perianal surgery and new intestinal complications

**Fig 3:**
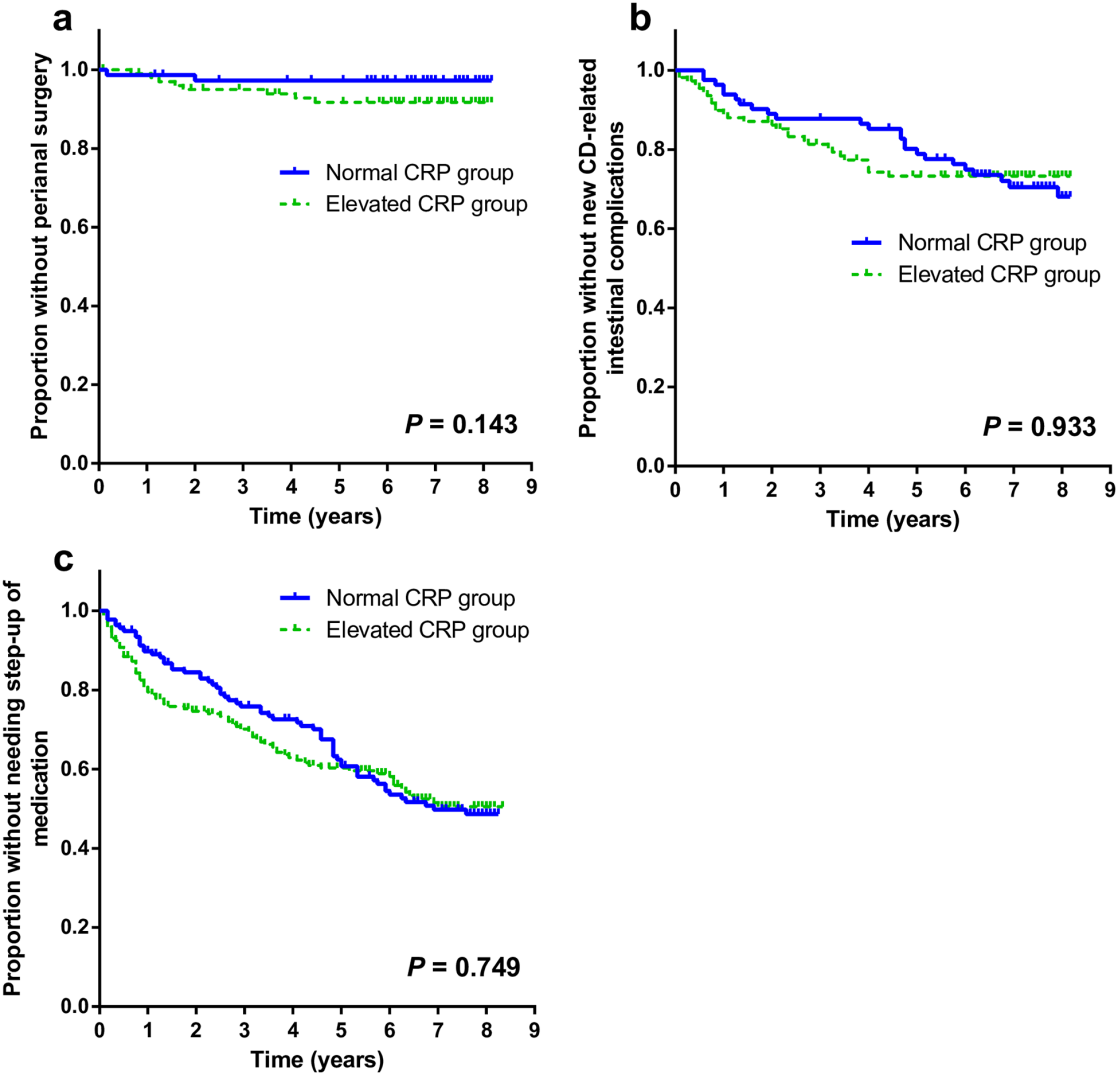
Kaplan–Meier new perianal surgery-free survival curves (a), new CD-related intestinal complication–free survival curves (b), and step-up of medication–free survival curves (c). CD, Crohn’s disease; CRP, C-reactive protein.

Among patients who did not experience prior perianal surgery at starting follow-up (n = 176), more patients required new perianal surgery in elevated CRP group than in normal CRP group (8 patients [7.8%] versus 2 patients [2.7%]). However, the Kaplan-Meier analysis with the log rank test showed no statistically significant difference (*P* = 0.143; Fig 3a) between two groups. Similarly, no significant difference was found between two groups regarding the development of new CD-related intestinal complications (Fig 3b).

### Step-up of medical therapy

After excluding 23 patients who had already been receiving anti-TNF-α therapy at beginning of follow-up, a total of 316 patients were analyzed for step-up in medical therapy. With regard to step-up of therapy, the Kaplan-Meier analysis with the log rank rest found no significant difference between two groups (*P* = 0.749; Fig 3c).

### Diagnostic test characteristics of elevated CRP in predicting outcome variables

The diagnostic test characteristics of elevated CRP for co-primary outcome variables (CD-related hospitalization and new CD-related intestinal resection) and secondary outcome variables (new perianal surgery, new CD-related intestinal complications, and step-up of medication) are shown in Table 5. Elevated CRP showed high negative predictive values for CD-related intestinal resection (92.2%). Also, elevated CRP showed substantial negative predictive values for CD-related hospitalization (79.4%) and new perianal surgery (74.8%), as well.

**Table 5:** Diagnostic test characteristics of elevated CRP in predicting outcome variables.

	Sensitivity	Specificity	Positive predictive value	Negative predictive value
CD-related hospitalization	33.9%	53.6%	13.3%	79.4%
CD-related intestinal resection	20.0%	56.6%	2.7%	92.2%
New perianal surgery	46.1%	58.9%	29.3%	74.8%
New CD-related intestinal complications	45.1%	56.1%	43.9%	57.3%
Step-up of medications	36.4%	50.7%	32.0%	55.6%

## Discussion

In this retrospective observational study, patients with CD in clinical remission showed a higher risk of subsequent CD-related hospitalization and CD-related intestinal resection if they had elevated CRP levels during period of clinical remission. To the best of our knowledge, there have been three studies which described the association between CRP level and prognosis in quiescent CD patients. André et al. found that raised CRP could predict relapse in asymptomatic patients with CD [17]. In addition, Boirivant et al. reported that elevated serum CRP was related to high relapse rate during follow-up [18]. In 2015, Click et al. reported that elevated CRP levels were associated with a higher risk of hospitalization in asymptomatic patients with CD [10]. Our findings are similar; however, our study is more robust. First, we assessed CD patients using CDAI score, which has been the gold standard for multiple clinical trials and studies [19]. Second, we used CDAI and CRP data measured two or more times over 6 months to ensure consistency in clinical status and laboratory values. Single Harvey–Bradshaw Index and CRP measurements might have caused misclassification bias in the earlier study [10]. Third, our study included a larger number of patients with elevated CRP levels (n = 189) compared with that in the previous study (n = 69), and we followed patients for a longer time (median, 7.9 years versus 2 years) [10].

We demonstrated that in CD patients in clinical remission, the risk of subsequent CD-related hospitalization was higher in patients with elevated CRP levels than in patients with normal CRP levels. The independent association between CRP level and subsequent CD-related hospitalization was also confirmed with the multivariate analysis. This result is consistent with the observation from the previous study, although hospitalization itself cannot be compared between studies due to differences in medical environment between the studies [10]. In our study, Montreal B3 behavior and perianal disease at baseline were also significantly associated with subsequent CD-related hospitalization, which is consistent with an observation in a prospective population-based study in an Australian cohort [20]. The association between perianal disease and further hospitalization risk was also observed in the study by Click et al. [10].

The new finding in this study is the association between elevated CRP and subsequent CD-related intestinal resection, which was a co-primary endpoint. Even during clinical remission, patients with elevated CRP levels had a higher risk of intestinal resection on follow-up, and this association was confirmed in the multivariate analysis as well. As observed in our study, B2 and B3 behaviors are well known as risk factors for intestinal resection [21–23].

The association between the use of immunomodulators and subsequent intestinal resection might suggest that patients with severe disease activity requiring immunomodulators have a higher risk of subsequent intestinal resection as well. However, the association between immunomodulator therapy and intestinal resection should be interpreted cautiously considering that data on duration and dosage of immunomodulator therapy was limited by our retrospective study design.

In this study, we found that CRP had a high negative predictive value for CD-related intestinal resection, CD-related hospitalization, and new perianal surgery. Therefore, if CRP level is maintained within normal range during clinical remission in patients with CD, favorable prognosis could be predicted and the frequency of unnecessary evaluation such as computed tomographic scan or endoscopy could be reduced.

CRP is a serum biomarker produced in the liver in response to several cytokines when inflammation is present in various tissues [24]. In CD, CRP levels reportedly reflect the inflammatory burden and can be used to predict short- and mid-term clinical flare-up [25]. Moreover, CRP levels show a correlation with endoscopic and histologic findings, which are reliable measures of CD activity [26]. Our findings suggest that similar to endoscopic or radiologic tools, elevated CRP may be a useful marker necessitating more detailed evaluations of intestinal inflammation even for CD patients in clinical remission.

Recent trends in CD management emphasize the progressive nature of the disease, which leads to cumulative structural damage regardless of symptoms [4]. Therefore, early diagnosis, early aggressive therapy, and close monitoring of disease activity are emphasized with the understanding that symptom-based treatment strategies are insufficient to modify the outcomes of disease progression such as intestinal resection [27, 28]. In line with this trend, mucosal healing is currently being emphasized as a treatment target because patients who achieve mucosal healing have a better prognosis than who do not [29, 30].

Recently, therapeutic targets of CD were proposed to achieve clinical/patient-reported outcome remission plus endoscopic/radiologic remission in the Selecting Therapeutic Targets in Inflammatory Bowel Disease (STRIDE) initiative [30]. In this initiative, biomarker remission (normal CRP and fecal calprotectin levels) was considered an adjunctive target, and it was strongly agreed that the failure of CRP or fecal calprotectin normalization should prompt further endoscopic or radiologic evaluation irrespective of symptoms [30]. CRP-guided endoscopic/radiologic evaluation and proper step-up therapy based on endoscopic/radiologic findings could control inflammation better and could result in mucosal healing and better prognoses [31, 32].

Our study has several limitations. First, although we prospectively enrolled CD patients in our IBD registry and actively managed our registry data according to the clinical status of the patients, the data for this study were retrospectively collected and analyzed. Second, we have not included information about other biomarkers such as fecal calprotectin, which was unavailable between 2008 and 2010 in our center. Compared with CRP, fecal calprotectin reportedly shows a stronger correlation with endoscopic findings [33], and evaluation of calprotectin together with CRP would have increased the quality of our study. Third, similar to the previous study, we did not mandate regimented clinical visits and monitoring at pre-specified time points as in prospective clinical trials [10]. However, on the contrary, our data reflect the natural disease course and true-to-life routine clinical care of CD patients, which makes our data collection more realistic than that in clinical trials, which usually exclude IBD patients in clinical remission. Finally, our study is based on data collected at a tertiary care center and may therefore have referral bias. However, in Korea, nearly all suspected CD patients are referred to tertiary hospitals for confirmatory diagnoses, and after the diagnosis of CD is confirmed, most patients are not referred back to private clinics and are instead retained at tertiary hospitals, which alleviates concern about referral bias.

## Conclusions

We found that among CD patients in clinical remission, those with elevated CRP had a higher risk of subsequent CD-related hospitalization and CD-related intestinal resection. Our findings suggest that even if patients with CD are in clinical remission, the presence of elevated CRP levels mandates additional evaluations of intestinal inflammation, which can lead to earlier and more aggressive therapeutic interventions and better clinical outcomes.

## References

[1] Abraham C, Cho JH. Inflammatory bowel disease. N Engl J Med. 2009; 361: 2066–2078. doi: 10.1056/NEJMra0804647 19923578 10.1056/NEJMra0804647 PMC3491806

[2] Baumgart DC, Sandborn WJ. Crohn's disease. Lancet. 2012; 380: 1590–1605. doi: 10.1016/S0140-6736(12)60026-9 22914295 10.1016/S0140-6736(12)60026-9

[3] Molodecky NA, Soon IS, Rabi DM, Ghali WA, Ferris M, Chernoff G, et al Increasing incidence and prevalence of the inflammatory bowel diseases with time, based on systematic review. Gastroenterology. 2012; 142: 46–54.e42; quiz e30. doi: 10.1053/j.gastro.2011.10.001 22001864 10.1053/j.gastro.2011.10.001

[4] Pariente B, Cosnes J, Danese S, Sandborn WJ, Lewin M, Fletcher JG, et al Development of the Crohn's disease digestive damage score, the Lemann score. Inflammatory bowel diseases. 2011; 17: 1415–1422. doi: 10.1002/ibd.21506 21560202 10.1002/ibd.21506 PMC3116198

[5] D'Haens G, Baert F, van Assche G, Caenepeel P, Vergauwe P, Tuynman H, et al Early combined immunosuppression or conventional management in patients with newly diagnosed Crohn's disease: an open randomised trial. Lancet. 2008; 371: 660–667. doi: 10.1016/S0140-6736(08)60304-9 18295023 10.1016/S0140-6736(08)60304-9

[6] Best WR, Becktel JM, Singleton JW, Kern F. Development of a Crohn's disease activity index. National Cooperative Crohn's Disease Study. Gastroenterology. 1976; 70: 439–444. 1248701

[7] Jones J, Loftus EV, Panaccione R, Chen LS, Peterson S, McConnell J, et al Relationships between disease activity and serum and fecal biomarkers in patients with Crohn's disease. Clin Gastroenterol Hepatol. 2008; 6: 1218–1224. doi: 10.1016/j.cgh.2008.06.010 18799360 10.1016/j.cgh.2008.06.010

[8] Modigliani R, Mary JY, Simon JF, Cortot A, Soule JC, Gendre JP, et al Clinical, biological, and endoscopic picture of attacks of Crohn's disease. Evolution on prednisolone. Groupe d'Etude Therapeutique des Affections Inflammatoires Digestives. Gastroenterology. 1990; 98: 811–818. 2179031 10.1016/0016-5085(90)90002-i

[9] Peyrin-Biroulet L, Reinisch W, Colombel JF, Mantzaris GJ, Kornbluth A, Diamond R, et al Clinical disease activity, C-reactive protein normalisation and mucosal healing in Crohn's disease in the SONIC trial. Gut. 2014; 63: 88–95. doi: 10.1136/gutjnl-2013-304984 23974954 10.1136/gutjnl-2013-304984

[10] Click B, Vargas EJ, Anderson AM, Proksell S, Koutroubakis IE, Ramos Rivers C, et al Silent Crohn's Disease: Asymptomatic Patients with Elevated C-reactive Protein Are at Risk for Subsequent Hospitalization. Inflammatory bowel diseases. 2015; 21: 2254–2261. doi: 10.1097/MIB.0000000000000516 26197446 10.1097/MIB.0000000000000516

[11] Hu PJ. Inflammatory Bowel Disease in Asia: The Challenges and Opportunities. Intest Res. 2015; 13: 188–190. doi: 10.5217/ir.2015.13.3.188 26130991 10.5217/ir.2015.13.3.188 PMC4479731

[12] Lee YJ, Yang SK, Byeon JS, Myung SJ, Chang HS, Hong SS, et al Analysis of colonoscopic findings in the differential diagnosis between intestinal tuberculosis and Crohn's disease. Endoscopy. 2006; 38: 592–597. doi: 10.1055/s-2006-924996 16673312 10.1055/s-2006-924996

[13] Loftus EV, Silverstein MD, Sandborn WJ, Tremaine WJ, Harmsen WS, Zinsmeister AR. Crohn's disease in Olmsted County, Minnesota, 1940–1993: incidence, prevalence, and survival. Gastroenterology. 1998; 114: 1161–1168. 9609752 10.1016/s0016-5085(98)70421-4

[14] Yang SK, Yun S, Kim JH, Park JY, Kim HY, Kim YH, et al Epidemiology of inflammatory bowel disease in the Songpa-Kangdong district, Seoul, Korea, 1986–2005: a KASID study. Inflammatory bowel diseases. 2008; 14: 542–549. doi: 10.1002/ibd.20310 17941073 10.1002/ibd.20310

[15] Park SH, Yang SK, Park SK, Kim JW, Yang DH, Jung KW, et al Long-term prognosis of crohn's disease and its temporal change between 1981 and 2012: a hospital-based cohort study from Korea. Inflammatory bowel diseases. 2014; 20: 488–494. doi: 10.1097/01.MIB.0000441203.56196.46 24412992 10.1097/01.MIB.0000441203.56196.46

[16] Satsangi J, Silverberg MS, Vermeire S, Colombel JF. The Montreal classification of inflammatory bowel disease: controversies, consensus, and implications. Gut. 2006; 55: 749–753. doi: 10.1136/gut.2005.082909 16698746 10.1136/gut.2005.082909 PMC1856208

[17] André C, Descos L, Vignal J, Gillon J. C-reactive protein as a predictor of relapse in asymptomatic patients with Crohn's disease. Scottish medical journal. 1983; 28: 26–29. doi: 10.1177/003693308302800106 6403982 10.1177/003693308302800106

[18] Boirivant M, Leoni M, Tariciotti D, Fais S, Squarcia O, Pallone F. The clinical significance of serum C reactive protein levels in Crohn's disease. Results of a prospective longitudinal study. Journal of clinical gastroenterology. 1988; 10: 401–405. 3418087 10.1097/00004836-198808000-00011

[19] Thia K, Faubion WA, Loftus EV, Persson T, Persson A, Sandborn WJ. Short CDAI: development and validation of a shortened and simplified Crohn's disease activity index. Inflammatory bowel diseases. 2011; 17: 105–111. doi: 10.1002/ibd.21400 20629100 10.1002/ibd.21400

[20] Niewiadomski O, Studd C, Hair C, Wilson J, Ding NS, Heerasing N, et al Prospective population-based cohort of inflammatory bowel disease in the biologics era: Disease course and predictors of severity. J Gastroenterol Hepatol. 2015; 30: 1346–1353. doi: 10.1111/jgh.12967 25867770 10.1111/jgh.12967

[21] Veloso FT, Ferreira JT, Barros L, Almeida S. Clinical outcome of Crohn's disease: analysis according to the vienna classification and clinical activity. Inflammatory bowel diseases. 2001; 7: 306–313. 11720320 10.1097/00054725-200111000-00005

[22] Peyrin-Biroulet L, Harmsen WS, Tremaine WJ, Zinsmeister AR, Sandborn WJ, Loftus EV. Surgery in a population-based cohort of Crohn's disease from Olmsted County, Minnesota (1970–2004). Am J Gastroenterol. 2012; 107: 1693–1701. doi: 10.1038/ajg.2012.298 22945286 10.1038/ajg.2012.298 PMC3572861

[23] Pandey A, Salazar E, Kong CS, Lim WC, Ong J, Ong DE, et al Risk of Major Abdominal Surgery in an Asian Population-based Crohn's Disease Cohort. Inflammatory bowel diseases. 2015; 21: 2625–2633. doi: 10.1097/MIB.0000000000000525 26240999 10.1097/MIB.0000000000000525

[24] Darlington GJ, Wilson DR, Lachman LB. Monocyte-conditioned medium, interleukin-1, and tumor necrosis factor stimulate the acute phase response in human hepatoma cells in vitro. J Cell Biol. 1986; 103: 787–793. 3017995 10.1083/jcb.103.3.787 PMC2114283

[25] Kiss LS, Papp M, Lovasz BD, Vegh Z, Golovics PA, Janka E, et al High-sensitivity C-reactive protein for identification of disease phenotype, active disease, and clinical relapses in Crohn's disease: a marker for patient classification? Inflammatory bowel diseases. 2012; 18: 1647–1654. doi: 10.1002/ibd.21933 22081542 10.1002/ibd.21933

[26] Solem CA, Loftus EV, Tremaine WJ, Harmsen WS, Zinsmeister AR, Sandborn WJ. Correlation of C-reactive protein with clinical, endoscopic, histologic, and radiographic activity in inflammatory bowel disease. Inflammatory bowel diseases. 2005; 11: 707–712. 16043984 10.1097/01.mib.0000173271.18319.53

[27] Bouguen G, Peyrin-Biroulet L. Surgery for adult Crohn's disease: what is the actual risk? Gut. 2011; 60: 1178–1181. doi: 10.1136/gut.2010.234617 21610273 10.1136/gut.2010.234617

[28] Sorrentino D. Preclinical and Undiagnosed Crohn's Disease: The Submerged Iceberg. Inflammatory bowel diseases. 2016; 22: 476–486. doi: 10.1097/MIB.0000000000000612 26752471 10.1097/MIB.0000000000000612

[29] Peyrin-Biroulet L, Ferrante M, Magro F, Campbell S, Franchimont D, Fidder H, et al Results from the 2nd Scientific Workshop of the ECCO. I: Impact of mucosal healing on the course of inflammatory bowel disease. J Crohns Colitis. 2011; 5: 477–483. doi: 10.1016/j.crohns.2011.06.009 21939925 10.1016/j.crohns.2011.06.009

[30] Peyrin-Biroulet L, Sandborn W, Sands BE, Reinisch W, Bemelman W, Bryant RV, et al Selecting Therapeutic Targets in Inflammatory Bowel Disease (STRIDE): Determining Therapeutic Goals for Treat-to-Target. Am J Gastroenterol. 2015; 110: 1324–1338. doi: 10.1038/ajg.2015.233 26303131 10.1038/ajg.2015.233

[31] Papi C, Fasci-Spurio F, Rogai F, Settesoldi A, Margagnoni G, Annese V. Mucosal healing in inflammatory bowel disease: treatment efficacy and predictive factors. Dig Liver Dis. 2013; 45: 978–985. doi: 10.1016/j.dld.2013.07.006 24018244 10.1016/j.dld.2013.07.006

[32] Baert F, Moortgat L, Van Assche G, Caenepeel P, Vergauwe P, De Vos M, et al Mucosal healing predicts sustained clinical remission in patients with early-stage Crohn's disease. Gastroenterology. 2010; 138: 463–468; quiz e410-461. doi: 10.1053/j.gastro.2009.09.056 19818785 10.1053/j.gastro.2009.09.056

[33] Schoepfer AM, Beglinger C, Straumann A, Trummler M, Vavricka SR, Bruegger LE, et al Fecal calprotectin correlates more closely with the Simple Endoscopic Score for Crohn's disease (SES-CD) than CRP, blood leukocytes, and the CDAI. Am J Gastroenterol. 2010; 105: 162–169. doi: 10.1038/ajg.2009.545 19755969 10.1038/ajg.2009.545

